# Supplementary material for: Explainable AI via learning to optimize
Source: Sci Rep. 2023 Jun 21;13:10103. doi: 10.1038/s41598-023-36249-3 (PMC10284861; doi:10.1038/s41598-023-36249-3)
Supplement: Supplementary file 1 — Supplementary Information. [file 41598_2023_36249_MOESM1_ESM.pdf]

Appendix: Explainable AI via Learning to Optimize

Howard Heaton[⋆†] **Samy Wu Fung**[⋆‡]

[†]Typal Research, Typal LLC
[‡]Department of Applied Mathematics and Statistics, Colorado School of Mines
research@typal.llc, swufung@mines.edu

A Certificate Code Snippet

The figure below shows how code for a `model` function can be tweaked to include certificates per standard software engineering practice.

Inference + Certificates → Trustworthy Inference

```
def TrustworthyInference(d):
    x, certs = model(d)
    if 'warning' in certs:
        warnings.warn('Warning Msg')
    if 'fail' in certs:
        raise Exception('Error Msg')
    return x
```

Figure 1: Example Python code to use certificates as post-conditions. Actual code should use specific warning/exception messages for flagged entries in `certs`.

B Linearized ADMM Formulation

Two of the numerical examples utilize variations of ADMM. This section is dedicated to a derivation of linearized ADMM used to solve problems of the form

$$\min_{x\in\mathbb{R}^n} f(Kx) + h(x) \ \text{ s.t. } \ \|Mx - d\| \leq \delta, \tag{1}$$

where K and M are linear operators, $\delta > 0$ is a noise tolerance, and f and h are proximable. First, we define the proximal operator for a closed, convex, and proper function by

$$\text{prox}_f(x) \triangleq \operatorname*{arg\,min}_{z\in\mathbb{R}^n} f(x) + \frac{1}{2}\|z - x\|^2. \tag{2}$$

Letting $\delta_{\mathcal{C}}$ be the indicator function for a closed and convex set $\mathcal{C}$ with value 0 for $x \in \mathcal{C}$ and ∞ otherwise, the Euclidean projection $\text{proj}_{\mathcal{C}}$ on $\mathcal{C}$ is a special case of the proximal, *i.e.*

$$\text{proj}_{\mathcal{C}}(x) \triangleq \text{prox}_{\delta_{\mathcal{C}}}(x) = \operatorname*{arg\,min}_{z\in\mathcal{C}} \frac{1}{2}\|z - x\|^2. \tag{3}$$

Next observe (1) can be rewritten as

$$\min_{x,w} f(Kx) + h(x) + \delta_{B(d,\delta)}(w) \quad \text{s.t.} \quad Mx - w = 0. \tag{4}$$

Defining the concatenation $\xi = (p, w)$, the function

$$g(\xi) \triangleq f(p) + \delta_{B(d,\delta)}(w), \tag{5}$$

and $S = [K; M]$, yields

$$\min_{x,\xi} h(x) + g(\xi) \quad \text{s.t.} \quad Sx - \xi = 0. \tag{6}$$

Then linearized ADMM[5] yields

$$x^{k+1} = \text{prox}_{\beta h}\left(x^k - \beta S^\top(\nu^k + \alpha(Sx^k - \xi^k))\right) \tag{7a}$$

$$\xi^{k+1} = \text{prox}_{\lambda g}\left(\xi^k + \lambda(\nu^k + \alpha(Sx^{k+1} - \xi^k))\right) \tag{7b}$$

$$\nu^{k+1} = \nu^k + \alpha(Sx^{k+1} - \xi^{k+1}), \tag{7c}$$

where α, β, λ are step-sizes. Rearranging, we obtain

$$\xi^{k+1} = \text{prox}_{\lambda g}\left(\xi^k + \lambda(\nu^k + \alpha(Sx^k - \xi^k))\right) \tag{8a}$$

$$\nu^{k+1} = \nu^k + \alpha(Su^k - \xi^{k+1}) \tag{8b}$$

$$x^{k+1} = \text{prox}_{\beta h}\left(x^k - \beta S^\top(\nu^{k+1} + \alpha(Sx^k - \xi^{k+1}))\right). \tag{8c}$$

Expanding ξ^{k+1} reveals block-wise updates, *i.e.*

$$p^{k+1} = \text{prox}_{\lambda f}(p^k + \lambda(\nu_1^k + \alpha(Kx^k - p^k)) \tag{9a}$$

$$w^{k+1} = P_{B(d,\varepsilon)}(w^k + \lambda(\nu_2^k + \alpha(Mx^k - w^k))), \tag{9b}$$

where $\nu^k = (\nu_1^k, \nu_2^k)$ and $P_{B(d,\varepsilon_\theta)}$ is the projection onto the Euclidean ball of radius ε_θ centered about d. Writing out expanded forms gives

$$p^{k+1} = \text{prox}_{\lambda f}\left(p^k + \lambda(\nu_1^k + \alpha(Kx^k - p^k))\right) \tag{10a}$$

$$w^{k+1} = P_{B(d,\delta)}\left(w^k + \lambda(\nu_2^k + \alpha(Mx^k - w^k))\right) \tag{10b}$$

$$\nu_1^{k+1} = \nu_1^k + \alpha(Kx^k - p^{k+1}) \tag{10c}$$

$$\nu_2^{k+1} = \nu_2^k + \alpha(Mx^k - w^{k+1}) \tag{10d}$$

$$x^{k+1} = \text{prox}_{\beta h}\left(x^k - \beta S^\top(\nu^{k+1} + \alpha(Sx^k - \xi^{k+1}))\right) \tag{10e}$$

Expanding the update for x^k reveals

$$x^{k+1} = x^k - \beta S^\top(\nu^{k+1} + \alpha(Sx^k - \xi^{k+1})) \tag{11a}$$
$$= x^k - \beta \begin{bmatrix} K \\ M \end{bmatrix}^\top \begin{bmatrix} \nu_1^{k+1} + \alpha(Kx^k - p^{k+1}) \\ \nu_2^{k+1} + \alpha(Mx^k - w^{k+1}) \end{bmatrix} \tag{11b}$$
$$= x^k - \beta K^\top \left(\nu_1^{k+1} + \alpha(Kx^k - p^{k+1})\right) \tag{11c}$$
$$- \beta M^\top \left(\nu_2^{k+1} + \alpha(Mx^k - w^{k+1})\right) \tag{11d}$$
$$= x^k - \beta K^\top \left(2\nu_1^{k+1} - \nu_1^k\right) \tag{11e}$$
$$- \beta M^\top \left(2\nu_2^{k+1} - \nu_2^k\right). \tag{11f}$$

The final form we implement is the tuple of update relations

$$p^{k+1} = \text{prox}_{\lambda f}\left(p^k + \lambda(\nu_1^k + \alpha(Kx^k - p^k))\right) \tag{12a}$$
$$w^{k+1} = P_{B(d,\delta)}\left(w^k + \lambda(\nu_2^k + \alpha(Mx^k - w^k))\right) \tag{12b}$$
$$\nu_1^{k+1} = \nu_1^k + \alpha(Kx^k - p^{k+1}) \tag{12c}$$
$$\nu_2^{k+1} = \nu_2^k + \alpha(Mx^k - w^{k+1}) \tag{12d}$$
$$r^k = K^\top\left(2\nu_1^{k+1} - \nu_1^k\right) + M^\top\left(2\nu_2^{k+1} - \nu_2^k\right) \tag{12e}$$
$$x^{k+1} = \text{prox}_{\beta h}\left(x^k - \beta r^k\right). \tag{12f}$$

C Supplement for Implicit Dictionary

The implicit dictionary problem described in the main draft is a special case of (1), taking $h = 0$, $M = A$, and $f = \|\cdot\|_1$. That is, in this case, we obtain the iteration

$$p^{k+1} = \eta_\lambda\left(p^k + \lambda(\nu_1^k + \alpha(Kx^k - p^k))\right) \tag{13a}$$
$$\nu_1^{k+1} = \nu_1^k + \alpha(Kx^k - p^{k+1}) \tag{13b}$$
$$\nu_2^{k+1} = \nu_2^k + \alpha(Ax^k - d) \tag{13c}$$
$$r^k = K^\top\left(2\nu_1^{k+1} - \nu_1^k\right) + A^\top\left(2\nu_2^{k+1} - \nu_2^k\right) \tag{13d}$$
$$x^{k+1} = x^k - \beta r^k, \tag{13e}$$

where η_λ is the shrink function.

D Supplement for CT Reconstruction

The CT reconstruction problem described in the main draft is a special case of (1), taking $h = \delta_{[0,1]^n}$, $M = A$, and $f = f_\Omega$. That is, in this case, we obtain the iteration

$$p^{k+1} = \text{prox}_{\lambda f_\Omega}\left(p^k + \lambda(\nu_1^k + \alpha(Kx^k - p^k))\right) \tag{14a}$$
$$w^{k+1} = P_{B(d,\delta)}\left(w^k + \lambda(\nu_2^k + \alpha(Ax^k - w^k))\right) \tag{14b}$$
$$\nu_1^{k+1} = \nu_1^k + \alpha(Kx^k - p^{k+1}) \tag{14c}$$
$$\nu_2^{k+1} = \nu_2^k + \alpha(Ax^k - w^{k+1}) \tag{14d}$$
$$r^k = K^\top\left(2\nu_1^{k+1} - \nu_1^k\right) + A^\top\left(2\nu_2^{k+1} - \nu_2^k\right) \tag{14e}$$
$$x^{k+1} = P_{[0,1]^n}\left(x^k - \beta r^k\right). \tag{14f}$$

TV minimization is obtained from CT reconstruction problem described in the main draft by letting f_Ω be the ℓ_1 norm and K be a discrete differencing operator. For comparison to an analytic method, we use anisotropic TV minimization, *i.e.*

$$\min_{u\in[0,1]^n} \|Du\|_1 \ \text{ s.t. } \ \|Au - d\| \leq \varepsilon, \tag{15}$$

where ε is hand-tuned. The Operator Discretization Library (ODL) Python library [1] is used to compute the filtered backprojections.

E Supplement for Cryptoasset Trades

This section is broken into three parts. The geometric constraint sets are of particular importance to handle in a decoupled fashion, and so the first subsection is dedicated to handling CFMM constraints for batches of transactions. Note closed form expressions exist for pairwise swaps. The second subsection then identifies the projection operations needed. This is followed by a derivation of a particular operator splitting used to solve the problem, giving explicit lists for updates.

Constraint Formulation

The dimension of the vector space for each CFMM may differ since some exchanges might not provide access to particular cryptoassets. Consequently, we follow similarly to recent work [2] in using matrices $A^j \in \mathbb{R}^{n_j \times n}$ to convert global coordinates into the local coordinates of the j-th CFMM, *i.e.*

$$A^j_{k\ell} \triangleq \begin{cases} 1 & \text{if token } k \text{ is in the } j\text{-th CFMM's coordinates} \\ & \text{is token } \ell \text{ is in global coordinates} \\ 0 & \text{otherwise.} \end{cases} \tag{16}$$

Note here we use the *backwards* of the referenced work, mapping global to local rather than local to global. Let $d \in \mathbb{R}^{n\times m}$ be a matrix with the j-th column d^j the reserve assets in the j-th CFMM. For weighted geometric CFMMs, set

$$\hat{d} \triangleq (1+\delta) \odot d \tag{17}$$

and

$$\alpha_j \triangleq \prod_{j=1}^{n_j} (\hat{d}_i^j)^{w_i^j}, \ \text{ for all } j \in [m], \tag{18}$$

where $\delta_j \geq 0$ is a tolerance, n_j is the number of asset types in the j-th CFMM, $w^j \in \mathbb{R}^{n_j}$ is a positive weighting, and

$$\mathcal{A}_j \triangleq \left\{ v \in \mathbb{R}^{n_j} : v + d^j \geq 0, \ \prod_{i=1}^{n_j} (v + d^j)^{w_i} \geq \alpha_j \right\}. \tag{19}$$

The set $\mathcal{A}_j$ identifies a weighted geometric mean inequality that must hold for the j-th CFMM. We include the nonnegative δ_j to account for noisy data. Choosing $\delta_j > 0$ gives a buffer for ensuring a transaction is still valid for noisy d (at the cost of reducing the achievable utility U).

Remark 1. *Ideally, we would directly compute $P_{\mathcal{A}_j}(x)$ in an algorithm computing optimal trades, which can be derived following an example in Beck's text.* [3] *However, this projection introduces unscalable coupling since, using $\delta_j = 0$ and $d = r$,*

$$[P_{\mathcal{A}_j}(x)]_i = \begin{cases} x_i & \text{if } x \in \mathcal{A}_j \\ \dfrac{x_i - r_i^j + \sqrt{(x_i + r_i^j)^2 + 4\lambda w_i^j}}{2} & \text{otherwise,} \end{cases} \tag{20}$$

where $\lambda > 0$ is a solution to

$$\sum_{i=1}^{n_j} w_i^j \log\left(\frac{x_i - r_i^j + \sqrt{(x_i + d_i^j)^2 + 4\lambda w_i^j}}{2}\right) = \log \alpha. \tag{21}$$

As the number of asset types in CFMMs increase, the time of a root finding algorithm to estimate λ also increases. Our alternative approach avoids this scaling issue. We also note JFB would technically require backpropping through the root finding scheme, but we suspect this could be avoided (by not attaching gradients during root finding) without adverse results.

Upon taking logarithms, we may equivalently write

$$\mathcal{A}_j = \left\{ v : v + d^j \geq 0, \ \sum_{i=1}^{n_j} w_i^j \ln(v_i + d_i^j) \geq \ln(\alpha_j) \right\} \tag{22a}$$

$$= \left\{ v : v + d^j \geq 0, \ \left\langle w, \ln(v + d^j) \right\rangle \geq \ln(\alpha_j) \right\}. \tag{22b}$$

We decouple the constraint $\mathcal{A}_j$ by defining the hyperplane

$$\mathcal{H}_j \triangleq \left\{ z : \left\langle w^j, z \right\rangle = \ln(\alpha_j) \right\}. \tag{23}$$

and the element-wise logarithm inequality constraint set

$$\mathcal{P}_j \triangleq \left\{ (v, z) : z \leq \ln(v + d^j), v + d^j \geq 0 \right\}. \tag{24}$$

These definitions yield the equivalence

$$v \in \mathcal{A}_j \iff \exists\, z \in \mathcal{H}_j \text{ s.t. } (v, z) \in \mathcal{P}_j. \tag{25}$$

This equivalence is useful since, as shown in a subsection below, $\mathcal{H}_j$ and $\mathcal{P}_j$ admit "nice" projection formulas. If instead the j-th CFMM is defined using a weighted arithmetic sum, then

$$\mathcal{A}_j = \left\{ x : x + d^j \geq 0, \left\langle w, x + d^j \right\rangle \geq \left\langle w, \hat{d}^j \right\rangle \right\} \tag{26a}$$

$$= \left\{ x : x + d^j \geq 0, \langle w, x \rangle \geq \left\langle w, \delta^j \odot d^j \right\rangle \right\}. \tag{26b}$$

Next define the Cartesian product

$$\mathcal{A} = \mathcal{A}_1 \times \cdots \times \mathcal{A}_m. \tag{27}$$

This enables the constraints to be expressed by

$$\mathcal{C}_\Theta(d) = \{(x, y) \geq 0 : A^j(\gamma_j x^j - y^j) \in \mathcal{A}_j \ \forall\, j \in [m]\}. \tag{28}$$

The tunable weights in $\mathcal{C}_\Theta(d)$ consist of the constraint tolerances δ_j. Let us introduce an auxiliary variable z and the block diagonal matrix $A = \text{diag}(A^1, \ldots, A^m)$. Additionally, let $\mathcal{I}_1 \subset [m]$ be the subset of CFMM indices with weighted geometric product constraints and $\mathcal{I}_2 \triangleq [m] - \mathcal{I}_1$ the remaining indices for weighted sum constraints. We obtain feasibility if and only if (v, x, y, z) is a minimizer of the sum of indicator functions

$$\delta_{\geq 0}(x) + \delta_{\geq 0}(y) + \delta_{\mathcal{R}}(v, x, y) \tag{29a}$$

$$+ \sum_{j \in \mathcal{I}_1} \delta_{\mathcal{P}_j}(v^j, z^j) + \delta_{\mathcal{H}_j}(z^j) + \sum_{j \in \mathcal{I}_2} \delta_{\mathcal{A}_j}(v^j), \tag{29b}$$

where

$$\mathcal{R} \triangleq \{(v, x, y) : v = \Gamma A x - A y\}. \tag{30}$$

This formulation of the constraints will be used in our operator splitting scheme.

Proximal/Gradient Operations

This section provides explicit formulas for the proximal and gradient operations needed. First note

$$P_{\geq 0}(x) = [x]_+ \triangleq \max(x, 0), \tag{31}$$

where the maximum occurs element-wise. The projection onto a hyperplane $\mathcal{H}_j$ is given by

$$P_{\mathcal{H}_j}(z) = z - \frac{\left\langle w^j, z - \ln(\hat{d}^j) \right\rangle}{\|w^j\|^2} w^j. \tag{32}$$

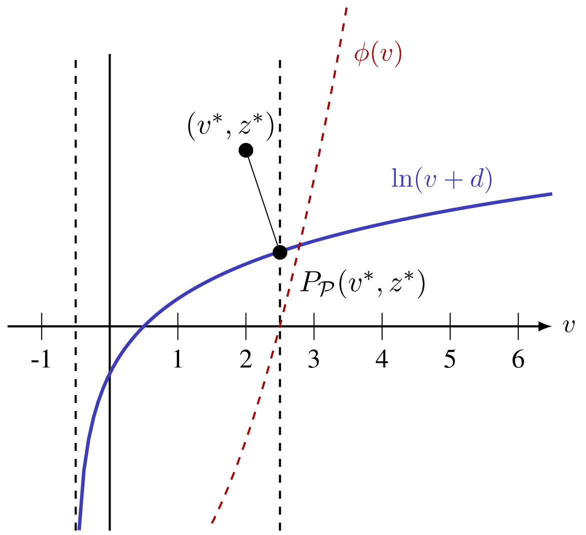

Supplementary Figure S1: Illustration for projection in $\mathbb{R}^2$ onto the set $\mathcal{P} \triangleq \{(v^*, z^*) : \ln(v + d) \geq z, v + d \geq 0\}$, which is all points below the blue curve $\ln(v + d)$. Here $d = 1/2$. The dashed red curve shows $\phi(v)$, the function defining the optimality condition for the projection in (35).

Similarly, if the j-th CFMM uses a weighted arithemtic,

$$P_{\mathcal{A}_j}(z) = z - \frac{[\langle w^j, z - \delta^j \odot d^j \rangle]_-}{\|w^j\|^2} w^j, \tag{33}$$

where $[z]_- \triangleq \min(z, 0)$. Next, the projection $P_{\mathcal{P}_j}$ is defined element-wise. The element-wise slope of $\ln(v + d)$ is $1/(v + d)$. The negative reciprocal of the slope (*i.e.* $-(v + d)$) gives the slope of the normal line passing through the projection and the point of interest. Letting $(\overline{v}^j, \overline{z}^j)$ be the projection of (v^j, z^j) gives the point-slope relation

$$\overline{z}^j - z^j = -(\overline{v}^j + d) \odot (\overline{v}^j - v^j). \tag{34}$$

Defining the function

$$\phi(v) \triangleq v \odot v + v \odot (d - v^j) - d \odot v^j + \ln(v + d) - z^j \tag{35}$$

enables the relation (34) can be expressed as

$$\phi(\overline{v}^j) = 0. \tag{36}$$

Since the above relation is element-wise and separable, each component $\overline{v}_i^j$ can be found independently (*e.g.* via a Newton iteration). We emphasize solving for each $\overline{v}_i^j$ is independent of the dimension n_j whereas computation costs for λ in (21) *increase* with n_j.

The final projection is for the linear constraint $\mathcal{R}$. The projection $P_{\mathcal{R}}(v, x, y)$ is a solution to the problem

$$\min_{(\overline{v}, \overline{x}, \overline{y})} \|\overline{v} - v\|^2 + \|\overline{x} - x\|^2 + \|\overline{y} - y\|^2 \ \text{ s.t. } \ \overline{v} = A(\Gamma \overline{x} - \overline{y}). \tag{37}$$

Let $N = [\Gamma A \ -A]$ and $\overline{q} = (\overline{x}, \overline{y})$ so the problem becomes

$$\min_{(\overline{v}, \overline{q})} \|\overline{v} - v\|^2 + \|\overline{q} - q\|^2 \ \text{ s.t. } \ \overline{v} = N\overline{q}. \tag{38}$$

It suffices to solve for $\overline{q}$ since the optimal $\overline{v}$ is then obtained by applying N. Substituting this in yields the simpler problem

$$\min_{\overline{q}} \|N\overline{q} - v\|^2 + \|\overline{q} - q\|^2, \tag{39}$$

for which the optimality condition is

$$0 = N^\top(N\overline{q}^\star - v) + \overline{q}^\star - q. \tag{40}$$

Rearranging gives the formula

$$\overline{q}^\star = (\mathrm{I} + N^\top N)^{-1}(q + N^\top v). \tag{41}$$

Letting

$$M \triangleq (I + N^\top N)^{-1} \tag{42}$$

and substituting in for $N^\top$ reveals

$$[P_{\mathcal{R}}(v, x, y)]_{(x,y)} = M \begin{bmatrix} x + A^\top \Gamma v \\ y - A^\top v \end{bmatrix}. \tag{43}$$

Lastly, we express the gradient for the utility U_Θ. Here

$$U_\Theta(x, y) = \sum_{j=1}^{m} \left\langle A^j p, A^j(y^j - x^j) \right\rangle \tag{44a}$$

$$- \frac{1}{2}\|W^j A^j(y^j - x^j)\|^2, \tag{44b}$$

where A^j is used to ensure the utility only measures cryptoassets that are available on the j-th CFMM (*i.e.* converts the global coordinates of x^j and y^j into the local coordinates of the CFMM), and each $W^j \in \mathbb{R}^{n_j \times n_j}$ penalizes transaction sizes in the j-th CFMM. For each j,

$$\nabla_{x^j} U_\Theta = -p - (W^j A^j)^\top (W^j A^j)(x^j - y^j) \tag{45}$$

and

$$\nabla_{y^j} U_\Theta = -\nabla_{x^j} U_\Theta. \tag{46}$$

Furthermore, U_Θ is L-Lipschitz with

$$L \triangleq \max_{j \in [m]} \|W^j A^j\|_2. \tag{47}$$

Operator Splitting Formulation

Set $\xi = (v, x, y, z)$ and define the functions

$$\delta_{\mathcal{M}}(v, z) \triangleq \sum_{j \in \mathcal{I}_1} \delta_{\mathcal{P}_j}(v^j, z^j) + \sum_{j \in \mathcal{I}_2} \delta_{\mathcal{A}_j}(v^j), \tag{48a}$$

$$\delta_{\mathcal{H}}(z) \triangleq \sum_{j \in \mathcal{I}_1} \delta_{\mathcal{H}_j}(z^j), \tag{48b}$$

where $\mathcal{M}$ and $\mathcal{H}$ are the sets corresponding to where the indicators in their definitions are all zero. Then define the functions

$$f(\xi) \triangleq \delta_{\geq 0}(x, y) + \delta_{\mathcal{M}}(v, z), \tag{49a}$$

$$g(\xi) \triangleq \delta_{\mathcal{R}}(v, x, y) + \delta_{\mathcal{H}}(z) \tag{49b}$$

$$h(\xi) \triangleq -U_\Theta(x, y). \tag{49c}$$

The informal crypto formulation in the main draft (20) may be equivalently expressed by

$$\min_\xi f(\xi) + g(\xi) + h(\xi), \tag{50}$$

where we note f and g are proximable and h is L-Lipschitz differentiable. We use Davis-Yin splitting[4] and $\alpha > 0$ iterate via

$$\xi^{k+1} = \mathrm{prox}_{\alpha f}(\zeta^k) \tag{51a}$$

$$\psi^{k+1} = \mathrm{prox}_{\alpha g}(2\xi^{k+1} - \zeta^k - \alpha \nabla h(\xi^{k+1})) \tag{51b}$$

$$\zeta^{k+1} = \zeta^k + \psi^{k+1} - \xi^{k+1}. \tag{51c}$$

For step size $\alpha \in (0, 2/L)$, we obtain the desired convergence $(\xi_x^{k+1}, \xi_y^{k+1}) \to (x_d, y_d)$.

References

[1] Adler, J.; Kohr, H.; and Öktem, O. 2017. Operator Discretization Library (ODL).

[2] Angeris, G.; Chitra, T.; Evans, A.; and Boyd, S. 2021. Optimal Routing for Constant Function Market Makers.

[3] Beck, A. 2017. *First-Order Methods in Optimization*. SIAM.

[4] Davis, D.; and Yin, W. 2017. A three-operator splitting scheme and its optimization applications. *Set-valued and variational analysis*, 25(4): 829–858.

[5] Ryu, E.; and Yin, W. 2022. *Large-Scale Convex Optimization: Algorithm Designs via Monotone Operators*. Cambridge University Press.
